# Oral carcinoma cuniculatum: an unacquainted entity with diagnostic challenges—a case report

**DOI:** 10.1186/s43046-021-00101-4

**Published:** 2022-01-17

**Authors:** Safaa Baz, Hatem Wael Amer, Ali A. Wahed

**Affiliations:** 1 Oral Pathology Department, Faculty of Dentistry, British University in Egypt (BUE), El Sherouk City, Suez Desert Road, P.O. Box 43, Cairo, 11837 Egypt; 2 Oral & Maxillofacial Pathology Department, Faculty of Dentistry, Cairo University, Cairo, Egypt

**Keywords:** Oral carcinoma cuniculatum, Cunniculate carcinoma, Rabbit burrows, Verrucous carcinoma, Oral squamous cell carcinoma, Differential diagnosis, Case report

## Abstract

**Background:**

Oral carcinoma cuniculatum (OCC) is an unacquainted well-differentiated subtype of oral squamous cell carcinoma, which displays unique clinic-pathological features. Up to date, OCC remains rare with about 75 reported cases, and is frequently missed or even misdiagnosed.

**Case presentation:**

The aim of the present work was: to report a case of OCC in mandibular gingiva and to highlight its main clinic-pathological diagnostic features: with an exophytic cobble-stone surface and a characteristic endophytic burrowing architecture, as well as to differentiate between it and other closely similar lesions including verrucous carcinoma, papillary squamous cell carcinoma, and well-differentiated conventional oral squamous cell carcinoma.

**Conclusions:**

An accurate diagnosis of OCC entails awareness of the clinicians and pathologists about its entity, proper knowledge of the diagnostic clinical and histopathological evidence, and the ability to differentiate it from closely similar lesions.

## Background

Carcinoma cuniculatum (CC), called cuniculate carcinoma, is a rare low-grade carcinoma [1]. Arid et al. reported a case series of CC on the plantar surface of the foot for the first time [2]. CC has been recorded in other sites including the abdominal wall, skin and, genital region [3–6]. However, in the oral cavity CC was first identified by Flieger and Owinski [7]. A plethora of synonyms were used in the past denoting this entity such as epithelioma cuniculatum, Buschke–Lowenstein tumor, and inverted verrucous carcinoma (VC) [1, 2, 5].

In terms of its unique clinical and pathological aspects, the World Health Organization (WHO) declared CC of the oral cavity, called oral carcinoma cuniculatum (OCC), as a separate well-differentiated subtype of oral squamous cell carcinoma (OSCC) in 2005 and 2017. Also, OCC and VC were considered as different subtypes, although of being confused in the past [1, 8]. OCC remains a rare entity that has diagnostic challenges, with a total of about 75 cases between 1977 and May 2021 were described in the literature [9]. Herein, we present a new case of OCC in the gingiva of an elderly female.

## Case presentation

**Fig. 1:**
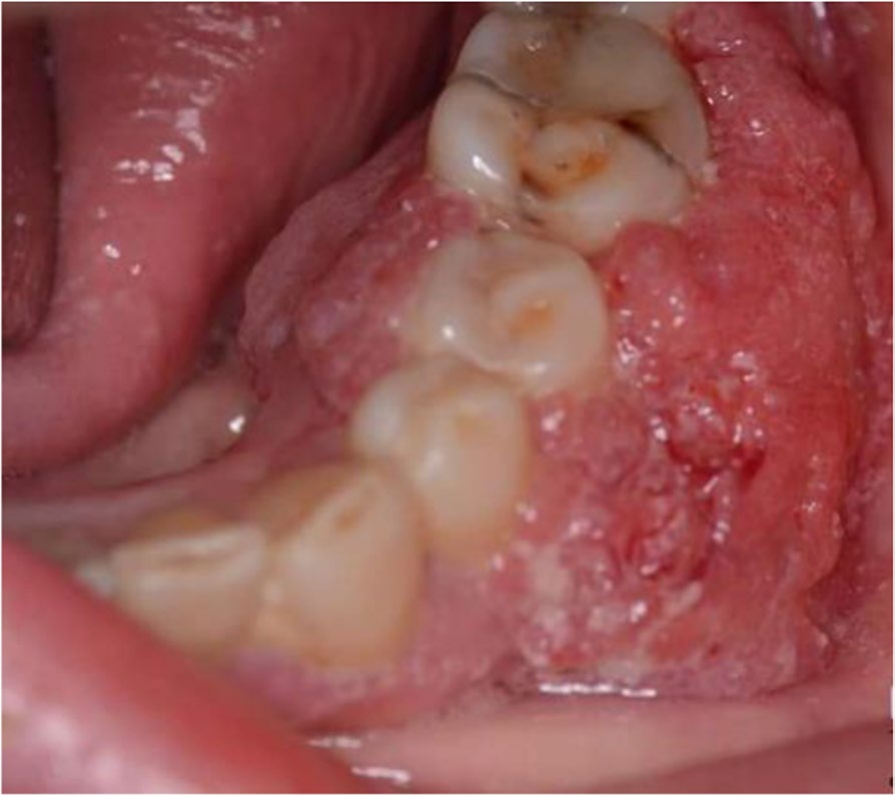
Clinical presentation of a pinkish and red-white colored lesion with an exophytic “cobble-stone” surface

An 86-year-old female patient presented with a 6 months history of non-painful lesion involved her left lower gingiva. A thorough medical history and clinical data were obtained. On intra-oral examination, a firm mandibular gingival swelling with blunt, pebbly “cobble-stone” surface was developed on both the buccal and lingual aspects of the premolar-molar region. The color of the lesion was similar to the surrounding normal mucosa intermixed with red-white areas (Fig. 1). No palpable draining lymph nodes were noted. Upon preoperative radiographic evaluation, no evidence of bone involvement was noted.

**Fig. 2:**
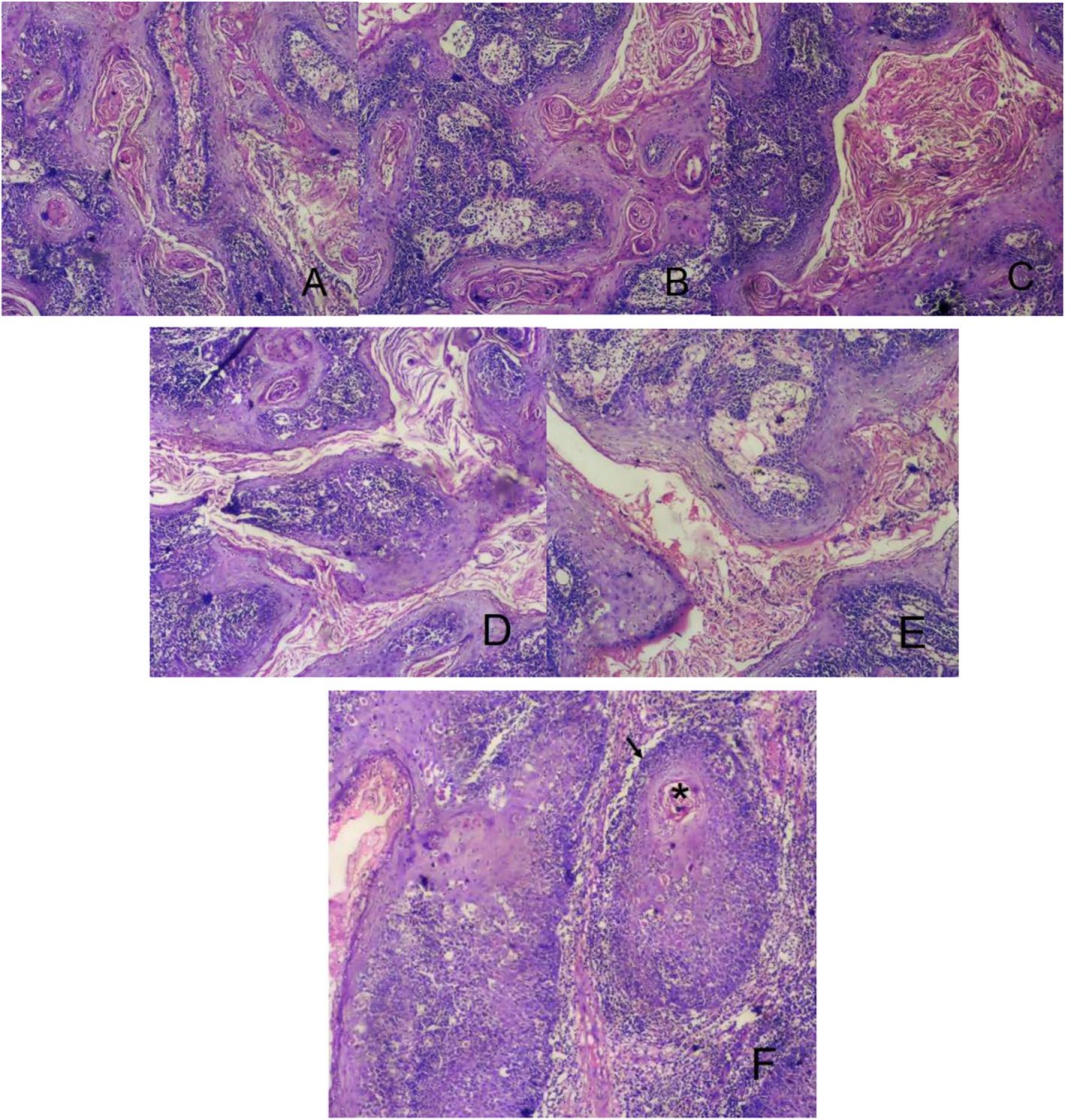
Photomicrographs, hematoxylin and eosin (H&E) staining, showing **A**, **B** endophytic deep “burrowing” growth of neoplastic epithelium with keratin-filled crypts (× 100). **C** A higher magnification (× 200). **D**, **E** Neoplastic cells forming cyst-like sinuses and tracts filled with keratin debris (× 100). **F** Neoplastic islands. Arrow points to the tumor nest and * indicates the keratin pearls (× 100)

An incisional biopsy specimen (measuring 1.5 × 1 cm down to bone) was obtained for histopathological examination. Microscopically, the lesion showed proliferation of the well-differentiated stratified squamous epithelium, forming endophytic complex branching networks interconnected with multiple deep keratin-filled clefts and crypts. Such characteristic “burrowing” architecture was evident, which resembles rabbit burrows; the hallmark of OCC (Fig. 2A–C). Besides, the neoplastic cells displayed tortuous cyst-like sinuses (Fig. 2D, E), and neoplastic islands of well-differentiated squamous cells showing minimal cellular atypia and mitosis, with stromal reaction. Notably, there was a close similarity of this field to the well-differentiated conventional OSCC (Fig. 2F).

Based on the previously mentioned clinicopathological features, a diagnosis of a separate variant of OSCC named OCC was made. The surgical decision was a wide excision as the case showed no bone invasion. Neck dissection was not carried out as there was no clinical indication. Of note, management was hard because of the patient’s age.

## Discussion

OCC is a unique variant of OSCC that shows distinct clinicopathological features [1, 8]. Intraorally, OCC occurs most commonly on mucoperiosteum, with a predilection for mandibular gingiva, as with our patient. Also, it can frequently affect the tongue. This tumor displays variable clinical presentations with/without bone involvement [10]. Unlike conventional OSCC, OCC differs mainly in that it has (1) a locally aggressive behavior with predominantly endophytic “burrowing” growth pattern, including branched sinuses and deep extending keratin-filled crypts and tracts that resemble rabbit burrows. Hence, the term “*cuniculatum”* derivates from the *Latin* meaning rabbit burrow crypts (rabbit-hole like) [11, 12], (2) a well-differentiated squamous epithelium with absent or minimal cellular atypia, and (3) a low-grade malignancy that has a better prognosis than conventional OSCC with a lesser tendency to metastasize. Nevertheless, it is crucial to note that the early diagnoses included metastases to local lymph nodes [13].

Unfortunately, its diagnosis is still challenging because OCC is frequently either missed or misdiagnosed and subsequently undertreated. The diagnostic dilemma of this lesion might return to the following factors:

- being unacquainted due to its low incidence in addition to insufficient knowledge about this entity, with consequent under-reporting [14].
- improper biopsy taking (fragmented, limited, or inadequate depth) which may omit the histopathological parameters necessary for reliable diagnosis [10].

**Fig. 3:**
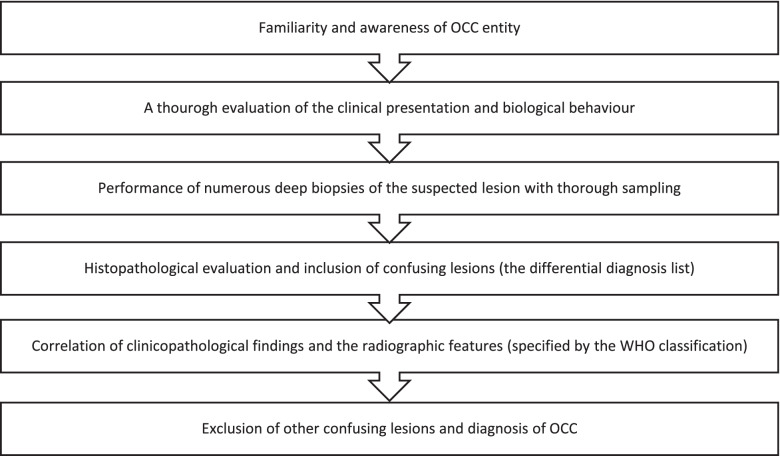
Basis for a proper approach to diagnose OCC

To avoid underdiagnosis of OCC and its confusion with other tumors, a diagnostic process is necessary which entails proper knowledge of its entity and diagnostic criteria, an extended clinical evaluation of the suspected cases, following by performance of proper biopsies, appropriate thorough specimen sectioning of these biopsies, and then careful histopathological assessment which is crucial to reveal the endophytic ‘burrowing pattern’, the hallmark of this lesion. Eventually, proper correlation of clinicopathological findings is essential for confident differentiation of OCC from other confusing lesions and in turn for its final diagnosis with the consequent establishment of the proper treatment plan (Fig. 3).

### Wide surgical excision for cases with no bone invasion

The differential diagnosis list of OCC includes lesions of the oral mucosa with exophytic surface and well-differentiated dysplastic epithelial component: VC, papillary squamous cell carcinoma (PSCC), and well-differentiated conventional OSCC. All of these lesions can be distinguished when biopsied, as each variant has a unique histopathological appearance. Consequently, a precise histological diagnosis can assist the clinician in planning accurate treatment, because the prognosis of each varies significantly. The distinctive properties of OCC and other similar lesions to differentiate between them are declared in Table 1.

**Table 1:** Differential diagnosis of oral carcinoma cuniculatum, verrucous carcinoma, papillary SCC, and well-differentiated conventional OSCC, respectively

	OCC	VC	PSCC	Well-differentiated OSCC
Histologic features				
Epithelium	Well-differentiated squamous epithelium	Well-differentiated squamous epithelium	Keratinizing type: dysplastic squamous epithelium non-keratinizing type: immature cells	Well-differentiated squamous epithelium
Cellular atypia	Absent or minimal (bland squamous cells)	Absent or minimal (bland squamous cells)	Severe (dysplastic squamous cells or immature basaloid cells)	Severe (dysplastic squamous cells)
Basement membrane	Invasive disruption of the basement membrane	Intact basement membrane with lateral spread	Invasive disruption of the basement membrane	Invasive disruption of the basement membrane
Growth pattern	Endophytic ± exophytic growth pattern predominantly endophytic growth, however both endophytic and exophytic growth can occur	Exophytic and endophytic growth pattern	Exophytic and endophytic growth pattern	Variable exophytic and/or endophytic growth pattern
Exophytic component	The surface is usually smooth but maybe exophytic. When exists the surface has a blunt pebbly ‘cobble stone’ appearance	Typically, the warty verrucous pattern with surface has vertical pointed ‘church spire-like’ fronds that show excessive surface keratin, clefting, and parakeratin plugging	The surface has a papillary ‘filiform’ pattern with thin central cores covered by malignant epithelial cells with minimal or no keratinization	When exists the surface has an irregular, fungating, papillary, or verruciform appearance
Endophytic component	Endophytic multiple deep burrowing ‘rabbit burrow’ or ‘cuniculi’ pattern of a complex arborizing network of invasive tumor cells with keratin-filled crypts and tortuous cystic structures into connective tissue is the hallmark of this lesion	The proliferating epithelium shows deeply invasive ‘pushing’ front of broad blunt elongated rete ridges and restricted to the lamina propria	The endophytic squamous proliferation of tumor cells into connective tissue	The endophytic squamous proliferation of tumor cells into connective tissue
	Infiltrating tumor islands	No infiltrating tumor islands	Infiltrating tumor islands	Infiltrating tumor islands
Invasion/infiltration	Locally destructive, infiltrative tumor	Superficial invasion	Locally destructive, infiltrative tumor	Aggressive locally destructive, infiltrative tumor
Keratin	Network of keratin-filled burrows and crypts in the connective tissue	Marked surface keratinization, so-called ‘church-spire keratosis’	Variable amount mostly confined to the surface	Abundant
	± Keratin pearls	Parakeratosis and parakeratin plugging	± Keratin pearls	++ Keratin pearls
Metastasis	Rare	Non-metastasizing	Uncommon	Frequent/early
Recurrence	Rare	Can recur	Can recur	Can recur
Prognosis	Worse prognosis than VC, but better prognosis than both PSCC and conventional OSCC	Better prognosis than OCC, PSCC, and conventional OSCC	Better prognosis than conventional OSCC	Worse prognosis than VC, OCC, and PSCC (According to clinical TNM^a^ staging)
Therapy	Wide surgical excision for cases with no bone invasion Subtotal maxillectomy or mandibulectomy with a safety margin for cases associated with bone invasion ± neck dissection in extensive cases	Wide surgical excision	Wide surgical excision + chemo- and/or radiotherapy	Stage I and II tumors are treated with *en bloc* resection ± neck dissection Stage III and IV tumors are treated with surgery with neck dissection + chemo- and/or radiotherapy

^a^ TNM: tumor (T), nodes (N), and metastases (M)

OCC managed for cases associated with bone involvement is subtotal maxillectomy or mandibulectomy with a safety margin. Meanwhile, wide surgical excision is the treatment of choice for cases with no bone invasion. This tumor is of low risk of metastasis. Neck dissection is only required if lymph node enlargement is clinically evident. The role of radiotherapy and chemotherapy remain questionable in the treatment of OCC [10].

## Conclusions

In conclusion, diagnosis of OCC requires familiarity with the entity, awareness of clinical and additional parameters, passing through obtaining abundant tissues, and ending with proper knowledge of the histopathological evidence that leads to an accurate diagnosis.

## Data Availability

The data used during the current study are available from the corresponding author on reasonable request.

## Abbreviations

CC: Carcinoma cuniculatum
VC: Verrucous carcinoma
WHO: World Health Organization
OCC: Oral carcinoma cuniculatum
OSCC: Oral squamous cell carcinoma
H&E: Hematoxylin and eosin
PSCC: Papillary squamous cell carcinoma
TNM: Tumor, nodes, and metastases
